# Low levels of Al stimulate the aboveground growth of *Davidia involucrata* saplings

**DOI:** 10.1186/s12870-024-05173-7

**Published:** 2024-05-28

**Authors:** Jun Wang, Jiong Guo, Houqi Yang, Xinqi Deng, Chunyan Zhang

**Affiliations:** 1 Key Laboratory of Southwest China Wildlife Resources Conservation (China West Normal University), Ministry of Education, Nanchong, 637009 Sichuan China; 2 Institute of Environmental Science, China West Normal University, Nanchong, Sichuan 637009 China; 3 College of Life Science, China West Normal University, Nanchong, Sichuan 637009 China

**Keywords:** Aluminum, Ecological strategies, Endangered species, Low pH

## Abstract

*Davidia involucrata* is a woody perennial and the only living species in the Genus *Davidia*. It is native to southern China where it holds cultural and scientific importance. However, *D. involucrata* is now an endangered species and its natural range includes low pH soils which are increasingly impacted by acid rain, nitrogen deposition and imbalanced nutrient cycling. The combination of these stresses also poses the additional risk of aluminum (Al) toxicity. Since the responses of *D. involucrata* to low pH and aluminum toxicity have not been investigated previously, a hydroponic experiment was conducted to examine the growth of one year old *D. involucrata* saplings after 50 d growth in a range of pH and Al conditions. Plant biomass, morphology, antioxidant enzyme activity, mineral concentrations and plant ecological strategy were compared at pH 5.8 and pH 4.0 without added Al (AlCl_3_) and in 0.1, 0.2 and 0.5 mM Al at pH 4.0. Our results showed that compared with pH 5.8, pH 4.0 (without added Al) not only inhibited root and shoot growth but also limited accumulation of nitrogen (N) and phosphorus (P) in leaves of *D. involucrate.* However, low Al concentrations (0.1 and 0.2 mM Al) at pH 4.0 partially restored the aboveground growth and leaf N concentrations, suggesting an alleviation of H^+^ toxicity by low Al concentrations. Compared with low Al concentrations, 0.5 mM Al treatment decreased plant growth and concentrations of N, P, and magnesium (Mg) in the leaves, which demonstrated the toxicity of high Al concentration. The results based on plant ecological strategy showed that *D. involucrate* decreased the competitiveness and favored its stress tolerance as pH changed from 5.8 to 4.0. Meanwhile, the competitiveness and stress tolerance of *D. involucrata* increased and decreased at low Al concentrations, respectively, and decreased and increased at high Al concentration, respectively. These trade-offs in ecological strategy were consistent with the responses of growth and antioxidant enzyme activity, reflecting a sensitive adaptation of *D. involucrata* to acid and Al stresses, which may aid in sustaining population dynamics. These findings are meaningful for understanding the population dynamics of *D. involucrata* in response to aluminum toxicity in acid soils.

## Introduction

*Davidia involucrata* Baill. (Family Davidiaceae) is a rare and endangered perennial tree species unique to China. This tree is a tertiary relict plant considered a “living fossil” and has been listed for priority state protection in the China Plant Red Data Book [1]. *D. involucrata* was once more widely distributed around the world but global climate changes in the Quaternary period decreased its distribution sharply. Currently, wild *D. involucrata* is largely restricted to the subtropical evergreen broad-leaved forests and the temperate deciduous broad-leaved forests of southwestern and south-central China [2, 3]. Reproduction and self-renewal are slow and difficult for this species and intensifying environmental stresses are decreasing its habitat further and increasing the risk of extinction [4]. Therefore, more study is required to understand the impact of specific environmental factors on fitness of *D. involucrata* to protect the remaining populations. Current research in this area mainly focuses on the impact of climate change factors such as temperature and water stress [1, 5, 6], while much less attention is being paid to soil factors.

As important limiting factors for plant growth and development, soil acidification and the accompanying increase of toxic aluminum (Al) are one of the important potential soil threats to the stability and sustainability of the wild population of *D. involucrata*. On the one hand, the distribution range of *D. involucrata* mainly comprises yellow and yellow-brown soils with pH values ranging from 4.0 to 6.5 [7, 8]. Furthermore, the soil acidification can be exacerbated by acid rain, nitrogen deposition, organic matter accumulation, and imbalanced soil nutrient cycling [9–11]. On the other hand, many soils in southwestern China also have high silicon and aluminum contents so that continued acidification can increase the concentration of Al in the soil solution, affect plant growth and threaten population stability [12]. Therefore, understanding the fitness status of *D. involucrata* under different levels of Al^3+^ treatment will help develop effective measures to protect the remaining *D. involucrata* populations. Unfortunately, until now, there has been no study clarifying how the fitness of *D. involucrata* will change under the threat of increased aluminum ions caused by soil acidification.

Generally, soluble Al^3+^ cations have negative effects on plant growth with the inhibition of root growth and changes in morphology being early symptoms of toxicity [13]. The resulting root systems are smaller and damaged and have a reduced capacity to take up water and nutrients [14, 15]. Previous studies have demonstrated that many woody perennials species in tropical and boreal forests are well adapted to acidic soils and can tolerate high concentrations of soluble Al [16]. In contrast to the high sensitivity exhibited of many crop species, these acid-soil adapted species are hardly affected by Al, and can even show a stimulation in growth by low to moderate concentrations of Al. This means the combination of low pH and Al can be either toxic or beneficial to plant growth depending on the species and conditions [9, 17, 18]. Thus, based on current information it is not possible to predict how low pH and Al affects the fitness of *D. involucrata* saplings.

The change in growth status is often regarded as an important reflection of the fate of plants in changing environments, but growth is actually a comprehensive reflection of trade-offs among three aspects of plant ecological strategy: resource acquisition, resistance to stress, and recovery after interference (such as regeneration and reproduction ability). These trade-offs provide an important insight into how the fitness of plant populations changes in changing environments. Because by deeply comparing the ecological strategies of plants in these three aspects, we can better understand when and how plants respond to environmental changes. Previous studies have attempted to explain these internal trade-offs in plants exposed to toxic Al often based on specific phenotypic, physiological or biochemical parameters [17, 19, 20]. However, due to the one-sided effect of individual parameters on physiological and metabolic processes, it is difficult to reveal the overall trade-offs of plant ecological strategies based solely on subjective indicators. Therefore, further quantitative and comprehensive understanding of the trade-offs of plant ecological strategies of *D. involucrata* under the changing Al concentrations may be more conducive to understanding of the fate of this rare and endangered plant under the threat of toxic Al in the future.

In order to understand the growth response and coping strategies of *D. involucrata* in relation to the increase of Al concentration under the acid condition, a hydroponic experiment with saplings of *D. involucrata* was conducted. Our objectives were to answer three questions: (1) Does low pH affect the growth of *D. involucrata* saplings? (2) Do different Al concentrations have different effects on the growth of *D. involucrata* saplings? (3) How do *D. involucrata* saplings alter their ecological strategies in response to different Al concentrations?

## Materials and methods

### Plant material, hydroponic culture and Al treatment

The one-year-old saplings of *D. involucrata* were obtained from a nursery (Yezhixin, Sichuan province) where the plants were grown from seed. After removal of the initial growth substrate, the roots were thoroughly rinsed with demineralized water. Then 40 saplings were randomly selected and transferred into 10 L tanks containing 8 L nutrient solution at pH of 5.8, with two saplings per tank, respectively. The nutrient solution consisted of (in µM) 625 KCl, 113 NH_4_Cl, 500 Ca (NO_3_)_2_, 125 MgSO_4_, 50 KH_2_PO_4_, 9 Fe-K_2_EDTA, 0.25 MnCl_2_, 0.02 CuSO_4_, 0.05 ZnSO_4_, 1.25 H_3_BO_3_, 0.005 Na_2_MoO_4_, and 0.00025 CoCl_2_ [21]. The nutrient solutions were changed weekly. The saplings were kept in this solution for two weeks to adapt to the hydroponic environment. After that, the 20 tanks were randomly divided into five groups with each group containing 4 tanks including 8 saplings. One group was randomly selected as control and treated with the above-mentioned nutrient solution at pH of 5.8. The other four groups were treated with the above-mentioned nutrient solution containing 0, 0.1, 0.2 and 0.5 mM AlCl_3_, respectively, at pH of 4.0. Al was added with vigorous stirring from a 1 M AlCl_3_ stock, freshly prepared on the day of use. The nutrient solutions were aerated vigorously by two air stones and aquarium pumps and renewed every three days.

These saplings of *D. involucrata* were cultivated in a naturally lit, and well-ventilated greenhouse with a day temperature range of 26–31 ^o^C, a night temperature range of 20–24 ^o^C, and a relative humidity range of 78–84%. Saplings were grown in these various treatments for 50 days.

### Growth measurements

Before the experimental treatments, the fresh plants were weighed (FW_before_) and the leaf numbers (LN_before_) were counted before they were transferred into the tanks. At the end of the experiments, the total fresh weight (FW_after_) and leaf numbers (LN_after_) of each plant were measured again. As a relative measure, fresh weight increment (ΔFW) was defined as: ΔFW = FW_after_ - FW_before_; leaf number increment (ΔLN) was defined as: ΔLN = LN_after_ - LN_before_.

After that, two relatively young but fully expanded leaves from the upper part of the plant were selected for determining leaf functional traits and plant ecological strategy. Another fully opened leaf of the terminal leaflets of the uppermost part of the plant was chosen for measuring antioxidant enzymes. The remainder of the leaves was scanned using a Portable Laser Area Meter (LI-3000 C, Li-Cor Inc., Lincoln, NE) for leaf area. The remainder of the leaves and the shoot were dried for 48 h at 70 °C and weighed for leaf dry weight and shoot dry weight. The dried leaves were used for plant elemental measurements. The total leaf area (TLA) and total aboveground dry weight (DW_ab_) were determined (including the leaves used for plant ecological strategy and antioxidant measurements).

The roots were scanned in a flatbed scanner (Epson Expression 10000XL) at a resolution of 400 dpi. The total root length, average root diameter and root volume were measured using WinRHIZO (Regent Instruments Inc., Quebec, QC, Canada). The new roots were separated and weighed after drying (RDW_new_). Similarly, the remainder of the roots were also dried at 70 °C for 48 h and weighed (RDW_re_). The total root dry weight (DW_root_) was determined as: DW_root_ = RDW_new_ + RDW_re_. The total individual dry weight (TDW) was determined as: TDW = DW_ab_ + DW_root_. The dry weight increment was calculated by the relative water content of the plant. The root/aboveground biomass ratio was calculated as DW_root_ divided by DW_ab_.

### Plant ecological strategy assessments

The plant ecological strategies of *D. involucrata* individuals were evaluated using the Competitor-Stress tolerator-Ruderal (CSR) ‘trade-off theory’ to explore the potential coping strategies of *D. involucrata* in response to Al treatments [22, 23]. According to the capacity of the plant in resource acquisition, stress tolerance and recovery after interference, life history strategy of plant can be defined as a trade-off in three primary strategies: competitive (C-strategy: more investment in large leaf or root size to acquire resources), stress tolerant (S-strategy: invest more resources into defensive traits or metabolic processes to protect tissue from stress damages), and ruderal (R-strategy: more investment in regeneration and reproduction). This plant ecological strategy ‘trade-off theory’ provides us a holistic perspective regarding the response of *D. involucrata* under different Al concentrations, especially when such CSR strategy trade-offs were quantified based on plant functional traits as reported by Pierce et al. (2017) [24].

According to the model reported by Pierce et al. (2017) [24], the CSR strategy trade-offs of *D. involucrata* individuals from different treatments were evaluated based on three fundamental traits: specific leaf area (SLA), leaf dry matter content (LDMC) and individual leaf area (LA), with the use of the spreadsheet calculation tool ‘StrateFy’. The shifting of coping strategies of *D. involucrata* along Al^3+^ concentrations gradient was analyzed by comparing the C, S and R percentage values of plant individuals under different treatments, respectively.

The SLA, LDMC and LA were measured according to the standardized methodologies detailed by Harguindeguy et al. (2013) [25]. The two leaves for plant ecological strategy measurements of each individual were scanned to obtain LA (scanned leaf area divided by leaf number, cm^2^), and then were immediately sealed in the plastic bags, saturated with water and CO_2_ by spraying water and breathing. The plastic bags were stored in a dark and cool box for a minimum of 12 to 24 h to achieve complete leaf turgidity. After that, these leaves were dried with soft paper towel and weighed for leaf water-saturated fresh weights. Leaf dry weight was recorded after dried at 70 °C for 48 h. Subsequently, SLA (leaf area/leaf dry weight, cm^2^g^− 1^) and LDMC (100 × leaf dry weight/leaf water-saturated fresh weight, %) were calculated.

### Antioxidant enzymes measurements

The leaf for antioxidant enzymes measurements of each individual was scanned and weighed to obtain leaf area and leaf fresh weight for total leaf area and total dry weight calculations. The collected leaves were homogenized with a mortar and pestle using liquid nitrogen. The total superoxide dismutase (SOD) activity was determined by measuring its ability to inhibit the photochemical reduction of nitroblue tetrazolium (NBT) [26, 27]. Peroxidase activity (POD) was measured according to the method of Chance and Maehly [28] with guaiacol as an electron donor, and the absorbance of the supernatant was determined at 470 nm. The activity was expressed in units of enzyme activity per minute per gram of tissue (min^− 1^ g^− 1^ FM). One enzyme unit was defined as the amount of enzyme causing an absorbance change of 0.1 min^− 1^ under standard conditions. The reaction of malondialdehyde (MDA) with thiobarbituric acid would produce a reddish-brown product under high temperature and acidic conditions, and determined by a spectrophotometer [20].

### Plant elemental measurements

The total leaf N concentration was measured by the semi-micro Kjeldahl method. The K, Ca and Mg concentrations of leaves and Al concentration of roots were determined by atomic absorption spectrophotometer [19]. The leaf P concentration was measured using an automatic intermittent chemical analyzer (Cleverchem 200) after digestion with HNO_3_ in a microwave oven [27].

### Statistical analyses

Data were analyzed using SPSS 19.0 software (SPSS Inc., Chicago, IL, USA). One-way ANOVAs were used to determine differences among treatments. For ANOVAs the data were tested for normality and equality of variations and, if necessary, natural log transformations were performed. Significant differences were determined at 95% significant level.

## Results

### Growth response

**Fig. 1:**
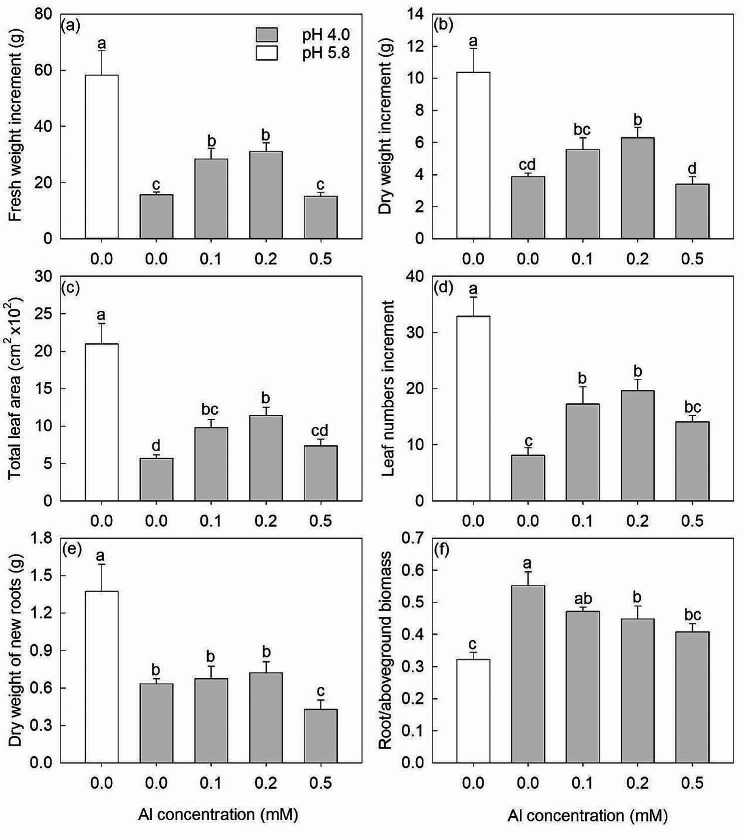
The fresh weight increment (**a**), dry weight increment (**b**), total leaf area (**c**), leaf number increment (**d**), dry weight of new root (**e**) and root/aboveground biomass ratio (**f**) under 0.0, 0.0, 0.1, 0.2 and 0.5 mM Al treatments. The white bars are pH 5.8 and the gray bars are pH 4.0. The data show means ± SE (*n* = 8). Different letters in the same sub-figure denote significant differences at the *P* < 0.05 level

Without added Al, the fresh weight increment, dry weight increment, total leaf area, leaf numbers increment and dry weight of new roots of the saplings became obviously lower, and the root/aboveground biomass ratio became significantly greater when the pH values changed from 5.8 to 4.0 (Fig. 1).

At pH 4.0, compared with 0 mM Al, the fresh weight increment, dry weight increment, total leaf area and leaf numbers increments were respectively 81% (*P* < 0.05), 44% (*P* = 0.14), 72% (*P* < 0.05) and 112% (*P* < 0.05) greater at 0.1 mM Al, and were respectively 98%, 62%, 101% and 143% greater at 0.2 mM Al (*P* < 0.05), but were not obviously changed at 0.5 mM Al (Fig. 1a-d). By comparison, the dry weight of new roots was not affected by 0.1 and 0.2 mM Al treatments, but showed a significant decrease at 0.5 mM Al (Fig. 1e). The root/aboveground biomass ratios at 0.1, 0.2 and 0.5 mM Al were 14% (*P* = 0.07), 20% (*P* < 0.05) and 27% (*P* < 0.05) lower than that at 0 mM Al, respectively (Fig. 1f).

### Root morphology

**Fig. 2:**
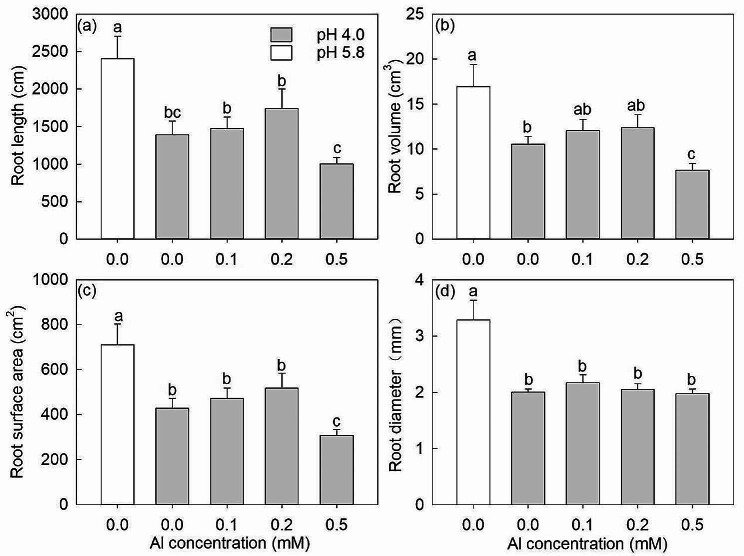
The root length (**a**), root volume (**b**), root surface area (**c**), and root diameter (**d**) under 0.0, 0.0, 0.1, 0.2 and 0.5 mM Al treatments. The white bars are pH 5.8 and the gray bars are pH 4.0. The data show means ± SE (*n* = 8). Different letters in the same sub-figure denote significant differences at the *P* < 0.05 level

Without added Al, the root length, root volume, root surface area and root diameter of *D. involucrata* saplings were all significantly lower at pH 4.0 than these values at pH 5.8 (Fig. 2). For the Al treatments at pH 4.0, root length, root volume and root surface area showed small increases (~ 20%) at 0.2 mM Al and significant decreases (~ 27%) at 0.5 mM Al compared with control 0 mM Al (Fig. 2a-c).

### Concentrations of N, P, K, ca, mg in the leaves and Al in the roots

**Fig. 3:**
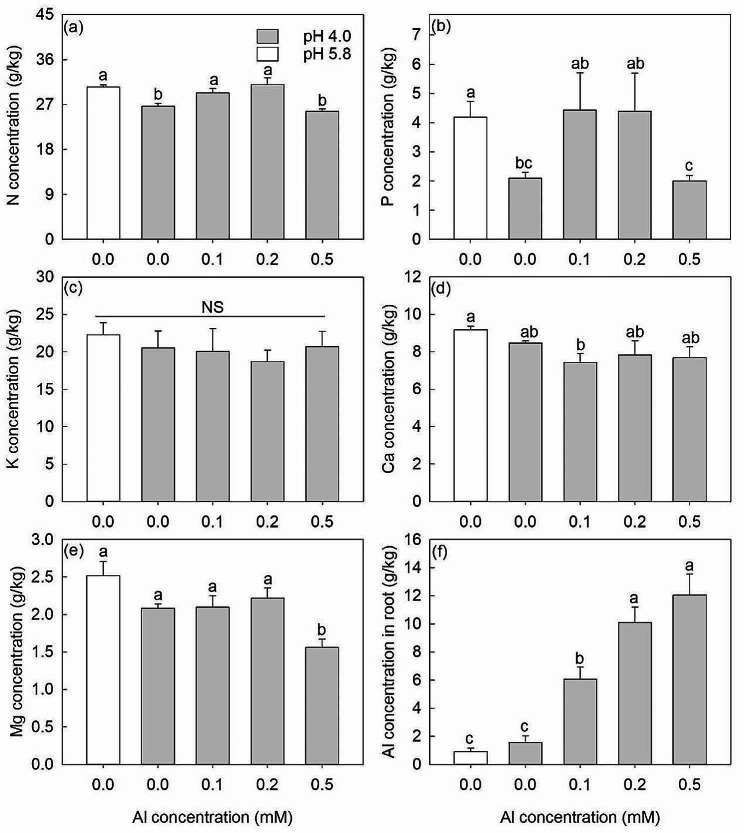
The N (**a**), P (**b**), K (**c**), Ca (**d**) and Mg (**e**) concentrations in leaves and Al (**f**) in roots under 0.0, 0.0, 0.1, 0.2 and 0.5 mM Al treatments. The white bars are pH 5.8 and the gray bars are pH 4.0. The data show means ± SE (*n* = 4). Different letters in the same sub-figure denote significant differences at the *P* < 0.05 level

The leaf N and P concentrations of saplings at pH 4.0 (0 mM Al) treatment were significantly lower than these values at pH 5.8 treatment. At pH 4.0, along with the increase of Al concentrations from 0 mM, the leaf N concentrations significantly increased by 10% and 16% at 0.1 and 0.2 mM Al treatments and leaf P concentrations increased but not significantly (*P* = 0.061) at 0.1 and 0.2 mM Al treatments. However, at 0.5 mM Al, both N and P concentrations decreased to values similar to those at 0 mM Al (Fig. 3a, b).

Leaf K and Ca concentrations were not affected by any Al treatment (Fig. 3c, d) while leaf Mg concentration showed a significant decrease at 0.5 mM Al compared with the other treatments (Fig. 3e). The Al concentration in the root tissues increased significantly as the external Al treatments increased in concentration (Fig. 3f).

### Antioxidant enzyme activity in the leaves

**Fig. 4:**
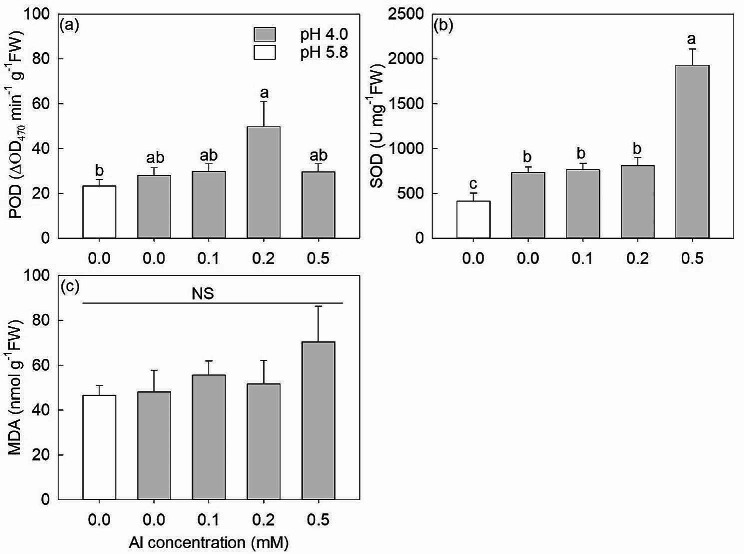
The peroxidase activity (POD) (**a**), superoxide dismutase activity (SOD) (**b**) and malondialdehyde content (MDA) (**c**) in leaves under 0.0, 0.0, 0.1, 0.2 and 0.5 mM Al treatments. The white bars are pH 5.8 and the gray bars are pH 4.0. The data show means ± SE (*n* = 5). Different letters in the same sub-figure denote significant differences at the *P* < 0.05 level

The pH 4.0 (0 mM Al) treatment had no effect on POD and significantly increased SOD compared to levels measured at pH 5.8 (Fig. 4a, b). POD and MDA content of leaves showed no significant changes with any Al treatment compared with the pH 4.0 (0 mM Al) treatment (Fig. 4a, c). By comparison, SOD showed a 2.5-fold increase at 0.5 mM Al treatment compared to other Al treatments (Fig. 4b).

### Leaf traits and plant ecological strategy

**Fig. 5:**
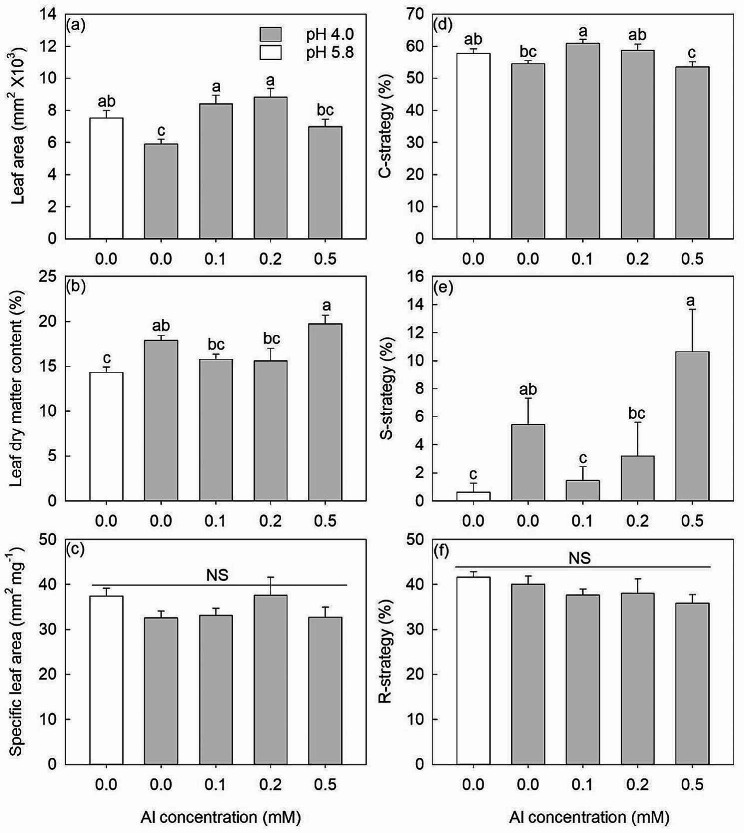
The three functional traits: leaf area (**a**), leaf dry matter content (**b**), and specific leaf area (**c**), along with Grime’s CSR strategies: C-strategy (**d**), S-strategy (**e**), and R-strategy (**f**) of *D. involucrata* saplings under 0.0, 0.0, 0.1, 0.2 and 0.5 mM Al treatments. The white bars are pH 5.8 and the gray bars are pH 4.0. The data show means ± SE (*n* = 8). Different letters in the same sub-figure denote significant differences at the *P* < 0.05 level

As shown in Fig. 5a and b, LA was significantly reduced and LDMC was significantly increased at pH 4.0 (0 mM Al) compared with the pH 5.8 treatment. When Al was added at pH 4.0, LA increased significantly at 0.1 and 0.2 mM Al by 42% and 49%, respectively, but decreased to a similar value as the 0 mM Al treatment at 0.5 mM Al. In contrast to the response of LA, compared with 0 mM Al treatment, LDMC displayed a decreasing trend at 0.1 and 0.2 mM Al and an increasing trend at 0.5 mM Al. Interestingly, compared to the 0.1 and 0.2 mM Al treatments, the 0.5 mM Al treatment significantly reduced LA and increased LDMC (Fig. 5a, b). Specific leaf area was not affected by any Al treatment (Fig. 5c).

According to the analysis using StrateFy, the *D. involucrata* saplings displayed a relatively weaker C-strategy and a significantly stronger S-strategy under pH 4.0 (0 mM Al) treatment compared to pH 5.8 treatment (Fig. 5d, e). At pH 4.0, compared with 0 mM Al treatment, the C-strategy increased by 11% (*P* < 0.05) and 7% (*P* = 0.058) in 0.1 and 0.2 mM Al while the S-strategy decreased by 73% (*P* < 0.05) and 41% (*P* = 0.068) at 0.1 and 0.2 mM Al treatments, respectively (Fig. 5d and e). By contrast, the 0.5 mM Al treatment significantly reduced C-strategy and greatly increased S-strategy, compared with 0.1 and 0.2 mM Al treatments (Fig. 5d, e). None of the pH or Al treatments had any effect on the R-strategy (Fig. 5f).

## Discussion

### Restricted growth of *D. Involucrata* under the acidic condition

Low pH has been reported to have various negative effects on morphology, physiology, and biomass accumulation in plants [29, 30]. In line with these previous reports, we found the dry weight of new roots and the increments of fresh weight, dry weight, leaf numbers and total leaf area of *D. involucrata* saplings were lower at pH 4.0 (0 mM Al) compared with pH 5.8.This suggested that the low soil pH condition, as well as the potential for further declines in soil pH value in the future, would pose a great threat to the growth of *D. involucrata*.

One of the reasons for this phenomenon of plant growth restriction caused by low pH may be that the increase of hydrogen ion concentration outside the roots inhibited the absorption of nutrients by the roots of *D. involucrata*, thus limiting the normal metabolism of the plants and the accumulation rate of organic matter. Our results showed that under the condition of pH 4.0, the leaf nitrogen and phosphorus contents were significantly lower than those under the pH 5.8 treatment. The study by Long et al. (2017) on *Citrus sinensis* and *Citrus grandis* also found that low pH not only significantly reduced the plant’s ability to absorb nutrients but also obviously inhibited the assimilation of carbon dioxide in the leaves [29]. According to previous studies at the plant cell level, the decrease in the amount of nutrient element absorption may be related to the inhibition of excess hydrogen ions on the function of plasma membrane H^+^-ATPase (PM H^+^-ATPase). Because PM H^+^-ATPase acts as a universal electrogenic H^+^ pump, and its function depends on the concentration gradient of H^+^ on both sides of the cell membrane. When the concentration of H^+^ in the environment is too high, this electrochemical proton gradient can be compromised, thereby limiting the transport of nutrients and other functions such as stomatal function [31].

The results also showed that the root/aboveground biomass ratio significantly increased, which indicated that aboveground plant properties responded more sensitively to low pH than root characteristics. This tendency of allocating more biomass underground implied a decrease in the efficiency of organic matter fixation through photosynthesis, per unit biomass, which also could be a significant factor contributing to the reduction in plant growth.

### Concentration-dependent effects of aluminum ion

This study clearly showed that under the condition of pH 4, low concentration Al could significantly promote the aboveground growth of *D. involucrata*. For example, although low levels of Al (0.1 and 0.2 mM Al) had no effect on dry weight of new roots and root characteristics, the increments of fresh weight, dry weight, leaf numbers and total leaf area of *D. involucrata* were almost all significantly higher than those at 0 mM Al. This is similar to the promotion effect of low concentration aluminum treatment on the growth of *Camellia japonica* and maize as found by Liu et al. 2020 [32] and Wang et al. (2015) [33], respectively. However, inconsistent with our results, Rehmus et al. (2014) found that a low Al dose of 0.3 mM enhanced the root biomass of *Tabebuia chrysantha* tree seedlings, but did not improve shoot biomass [17]. This inconsistency may be attributed to the variations in the concentrations of nutrients in different organs. As suggested by Rehmus et al. (2014), the higher P concentrations in roots than those in the leaves may result in the root biomass increase at low concentration Al [17]. By comparison, our finding showed a higher N concentration in leaves of *D. involucrata* saplings at low Al concentrations (0.1 and 0.2 mM) compared with 0 mM Al treatment (pH 4.0). Increased N concentrations were likely beneficial to plant growth because N is necessary for protein synthesis. Therefore, as reported by Wang et al. (2015) who found a low Al concentration increased leaf protein content and thus promoted leaf growth [33], the increased leaf N concentrations may be one reason for the increased aboveground growth of *D. involucrata* under low pH.

This enhanced N concentration at low Al concentrations might be explained by alleviation of H^+^ toxicity. Previous studies showed that at low pH, low concentrations of Al could displace the highly toxic H^+^ ions from critical binding sites in the cell wall and on the plasma membrane [34]. They could also enhance *PM H*^*+*^*-ATPase* expression, thereby promoting H^+^ extrusion [35]. The enhanced PM H^+^-ATPase activity may regulate the function of nutrient transporters, such as ammonium transporters, to promote NH_4_^+^ uptake [36]. Because NH_4_^+^ uptake and assimilation are closely synchronized, the NH_4_^+^ assimilation would provide amino acids for protein synthesis and growth [36].

Our study also demonstrated that the growth of *D. involucrata* was inhibited by high Al concentration. For example, when the Al concentration was increased to 0.5 mM, the increments of fresh weight, dry weight, leaf numbers and total leaf area of the *D. involucrata* reduced to values similar to those of the 0 mM Al treatment. Moreover, consistent with the results found by Ryan et al. (2001) [37]and Hiranoet al. (2012) [38], the root growth and root traits of *D. involucrata* treated with 0.5 mM Al were worse than those treated with 0 mM Al. The inhibited absorption of nutrient elements may be one of the main reasons for the restricted growth of *D. involucrata* under high concentration aluminum treatment. Because we also found that under 0.5 mM Al treatment, leaf N and P concentrations decreased to the same level as under 0 mM Al treatment, and the leaf Mg concentration decreased to a significantly lower level than that under 0 mM Al treatment. The limited nutrient absorption capacity may be attributed to the reason that excess Al ions bind to the negatively charged phospholipid bilayers of the plasma membrane, which destabilizes the membrane potential and impedes the H^+^-ATPase’s ability to exclude protons, thereby affecting the transport of nutrients [39–41].

This concentration-dependent effect of Al suggested that low Al concentration in an acid environment may reduce the negative effect of low pH on the growth of *D. involucrata*. For the protection of *D. involucrata* population under the trend of soil acidification, we should be vigilant about the potential negative impacts of soil with high Al concentration on its growth.

### Different coping strategies of *D. Involucrata* in response to acid and aluminum environments

Whether plants can cope with adverse environments through ecological strategy adjustments is the key to maintain their fitness as much as possible in stressful environments [42–44]. Our finding showed that *D. involucrata* saplings had a significantly greater S-strategy at pH 4.0 compared to pH 5.8, but had a relatively lower C-strategy, which is in line with the decreased biomass and increased SOD activity. These results indicated that under the acid condition, *D. involucrata* saplings adopted a trade-off strategy of reducing growth and enhancing resistance, thereby improving their survival ability.

Compared with 0 mM Al treatment (pH 4.0), C-strategy of *D. involucrata* saplings increased and S-strategy decreased at low Al concentrations (0.1 and 0.2 mM), but decreased and increased, respectively at 0.5 mM Al concentration. These changes were consistent not only with biomass increase at low Al concentrations (0.1 and 0.2 mM) and biomass decrease at 0.5 mM Al concentration, but also with the response of resistance physiological indicators. For instance, we found activation of antioxidants (POD and SOD) of *D. involucrata* saplings did not change at low Al concentrations, but SOD activity increased at 0.5 mM Al treatment. The trade-off between growth and resistance was also in line with the biochemical responses observed in previous studies. Specifically, under high Al stress, plants not only increased energy expenditure to enhance activation of antioxidants, but they also devoted more energy to exude organic acids or phenolic compounds [45]. These would alleviate Al toxicity within the plant or reduce Al concentration in the rhizosphere [14, 46, 47].

The strategic adjustments observed in *D. involucrata* suggested that, in response to acid and aluminum stress, the plant could modify its energy and resource allocation between growth and resistance. This adaptation provided an additional explanation for changes in growth patterns under varying Al conditions and might aid in sustaining population continuity. We believe that further research on the transformation of ecological strategies, as well as the Al concentration threshold for effective defense strategies, taking into account metabolic and gene expression changes, will benefit further understanding and predicting the population dynamics of *D. involucrata* under the worsening crisis of acid and aluminum stress.

## Conclusions

The present study demonstrated that low pH (pH 4.0) inhibited the growth of *D. involucrata* saplings, and while low Al concentrations could alleviate the negative effects of low pH, as the Al concentration increased, the inhibitory effects on the growth were once again enhanced. Compared with pH 5.8, *D. involucrata* saplings adopted a trade-off strategy of reducing their competitiveness and increasing its resistance at pH 4.0. At pH 4.0, C-strategy and S-strategy were greater and lower respectively under low Al concentrations than those under 0 mM Al treatment, but as the Al concentration increased to 0.5 mM, these two strategies significantly decreased and increased, respectively. Our results illustrated that (1) the acid condition alone limited the growth of *D. involucrate* saplings, and the influence of Al depended on the concentration; (2) under the different aluminum treatments, *D. involucrata* can flexibly adjust its trade-off between competitive and stress tolerance strategies, which may better maintain its fitness and population continuation. Further studies on the growth changes and ecological strategies trade-off within a larger range of Al concentrations, along with research on related metabolic and gene expression processes, will benefit attempting to further explain and predict the dynamics of *D. involucrata* population under the Al stress.

## Data Availability

All data generated or analysed during this study are included in this published article.
